# Through the looking glass: Confronting health care management’s biggest challenges in the wake of a crisis

**DOI:** 10.1097/HMR.0000000000000365

**Published:** 2023-02-16

**Authors:** Rachel Gifford, Frank van de Baan, Daan Westra, Dirk Ruwaard, Bram Fleuren

**Affiliations:** **Rachel Gifford,** PhD, Assistant Professor, Department of Health Services Research, Care and Public Health Research Institute, Faculty of Health, Medicine and Life Sciences, Maastricht University, the Netherlands. E-mail: r.gifford@maastrichtuniversity.nl.; **Frank van de Baan,** MSc, PhD Candidate, Department of Health Services Research, Care and Public Health Research Institute, Faculty of Health, Medicine and Life Sciences, Maastricht University, the Netherlands.; **Daan Westra, PhD,** Assistant Professor, Department of Health Services Research, Care and Public Health Research Institute, Faculty of Health, Medicine and Life Sciences, Maastricht University, the Netherlands.; **Dirk Ruwaard, MD, PhD,** Professor and Departmental Chair, Department of Health Services Research, Care and Public Health Research Institute (CAPHRI), Faculty of Health, Medicine and Life Sciences, Maastricht University, the Netherlands.; **Bram Fleuren, PhD,** Assistant Professor, Department of Work and Social Psychology, Faculty of Psychology and Neuroscience, Maastricht University, the Netherlands.

**Keywords:** Hospitals, healthcare managers, human resources, qualitative methods

## Abstract

The challenges brought on by the pandemic triggered a renewed scholarly focus on managing during crises. Now, 3 years on, having covered the initial crisis response, it is important to reevaluate what the crisis has taught us about health care management more generally. In particular, it is useful to consider the persistent challenges that continue to face health care organizations in the wake of a crisis.

**Purpose:**

The present article aims to identify the biggest challenges that currently face health care managers in order to formulate a postcrisis research agenda.

**Methodology/Approach:**

We employ an exploratory qualitative study, utilizing in-depth interviews with hospital executives and management to explore the persistent challenges facing managers in practice.

**Results:**

Our qualitative inquiry reveals three key challenges that extend beyond the crisis and are salient for health care managers and organizations in the years to come. Specifically, we identify the centrality of human resource constraints (amidst increasing demand), the necessity of collaboration (amidst competition), and a need to reconsider the approach to leadership (utility of humility).

**Conclusion:**

We conclude by drawing upon relevant theories such as paradox theory to formulate a research agenda for health care management scholars that can support the creation of novel solutions and approaches to persistent challenges in practice.

**Practice Implications:**

We identify several implications for organizations and health systems, including the need to eliminate competition and the importance of building human resource management capacities within organizations. In highlighting areas for future research, we provide organizations and managers with useful and actionable insights to address their most persistent challenges in practice.

Health care organizations (HCOs) play a substantial role in addressing grand societal challenges (Hefner & Nembhard, 2021; Mayo et al., 2021). The COVID-19 pandemic constitutes the most recent and, arguably, currently the most urgent example of such a challenge (Hefner & Nembhard, 2021). In the onset of the pandemic, scholars quickly synthesized prior (health care) management literature to offer insights and strategies that could help health care managers and systems cope with and respond to the challenges the crisis brought on (e.g., Nembhard et al., 2020). However, as HCOs progress through the third year of the COVID-19 pandemic, the field has been significantly altered, and our strategies require updating. In addition, it has been recognized that the work of (health care) management researchers typically remains too disconnected from the most pressing needs of (health care) managers (Alexander et al., 2007; Fainshmidt et al., 2021). For example, the lack of emphasis on the level of and strategies for managers within HCOs and their role in addressing pressing grand challenges or wicked problems (Fainshmidt et al., 2021) perpetuates a mismatch between theorizing and practice. It is thus imperative that we empirically assess the challenges health care managers face in practice and calibrate research efforts accordingly.

A burgeoning body of literature has rapidly sought to address the most salient and pressing challenges brought on by the COVID-19 crisis. Effective policy responses (e.g., Alami et al., 2021; Courtemanche et al., 2020; Haldane et al., 2021; Leung et al., 2021) and the well-being of health care workers (e.g., Fleuren et al., 2021; Lai et al., 2020) constitute two key challenges that health services and health care management researchers have studied in response to the pandemic. The former body of literature focuses on the various policy responses enacted by national and/or local policymakers in response to the pandemic and their effectiveness in terms of reducing infection and mortality rates (Courtemanche et al., 2020; Leung et al., 2021). The latter has clearly illustrated the consequences associated with the increased pressure the pandemic has put on frontline health care professional (e.g., Lai et al., 2020) and has begun to unravel the key mechanisms behind this process (e.g., Fleuren et al., 2021). Both of these streams of literature address issues that are of great concern to contemporary health care managers but may not fully capture the broad array of challenges they face (Gifford et al., 2022; Nembhard et al., 2020) as they continue through and, most importantly, beyond the pandemic.

Now that the crisis has evolved through several phases, there is a need for both retrospection and prospection. In this article, we draw upon a qualitative study to empirically asses the persistent challenges of health care management in the present day, working to create an agenda that extends beyond the context and specificities of the crisis. Persistent challenges are defined here as challenges that managers and health care leaders identify as existing prior to and through (or exacerbated by) the pandemic. Drawing upon in-depth interviews with executives and management across multiple hospitals, we explore the persistent challenges facing managers in practice and draw upon what the crisis has taught them about management to expand upon initial scholarly insights. Rather than considering how management can take action *during* a crisis, we argue it is important to consider what the context of a crisis can teach us about management going forward (see Muzio & Doh, 2021). Consequently, the present study provides two central contributions: (a) to unveil the persistent challenges facing health care management in the wake of a crisis and (b) to establish a research agenda based on these challenges.

## Theory

The COVID-19 crisis constitutes a disruptive event that has upended and significantly impacted the field of health care with effects that will reverberate for years to come (Haldane et al., 2021; Muzio & Doh, 2021). However, such an event also opens up unique opportunities for health care management scholars to expand the bounds of our current theorizing. In the aftermath of significant disruption, it is therefore important to recalibrate the field and update our current theorizing to ensure our theories remain responsive and connected to the realities and demands of practice (Fainshmidt et al., 2021). In the following sections, we begin by identifying key themes that were prevalent in emergent work in the onset of COVID-19. Although it is beyond the scope of the present article to provide an overview of key themes in health care management more generally, three recent reviews of the field offer useful insights in this regard (Mayo et al., 2021; Reay et al., 2021; Zengul et al., 2022). We conclude by emphasizing the need for more empirical work to move beyond COVID-19-specific strategies and help guide the formulation of a research agenda to meet our most pressing challenges for the future.

### Managing in Crisis: Salient Topics for Health Care Management

In the onset of the COVID-19 crisis, health care leaders were faced with complex challenges for which there was scarce evidence to support their responses (Park et al., 2020). Various scholars worked to offer timely insights for health care managers and leaders to help them respond to and remain resilient in the face of crisis (Begun & Jiang, 2020; Forman et al., 2020; Haldane et al., 2021; Hick & Biddinger, 2020; Nembhard et al., 2020; Park et al., 2020). For example, Nembhard et al. (2020) surveyed the management literature to offer a list of actions for health care managers to respond to the COVID-19 crisis. Their lessons include putting people first, implementing systems thinking and creativity, focusing on relational coordination and teamwork, forming interorganizational and cross-sector partnerships, and conveying clarity and humility in leadership. Similarly, Begun and Jiang (2020) argue that communication, collaboration, and innovation are essential for HCOs’ response to the pandemic. Hick and Biddinger (2020) adopt a more medically oriented perspective but stress the importance of protecting workers, adjusting operations to sudden changes, collaboration across-sectors, and the role of innovative information technology and artificial intelligence solutions (see Table 1 for an overview of select studies that offer insights for health care managers at the organizational level during crisis).

**Table 1:** Overview of lessons and proposed strategies for health care managers and health system leaders (synthesis of existing reviews)

		Organizational level			Health system level		
Theme	Category	Nembhard et al. (2020)	Begun & Jiang (2020)	Hick & Biddinger (2020)	Forman et al. (2020)	Alami et al. (2021)	Haldane et al. (2021)
Resources	Human resources	Putting people first	Communication	Protecting health care workers	—	Promote health and well-being of professionals	Training and well-being of health care workforce
	Operations	Systems thinking and creativity	—	Adjusting to surges and reductions in demands for services	—	—	—
	Innovation	—	Ambidexterity	Use of artificial intelligence and information technology solutions to support organizations	Need for novel artificial intelligence and robotics solutions	Telehealth and digital health	—
Collaboration	Team dynamics	Relational coordination and teamwork	—	—	—	Interprofessional work and interdisciplinarity	—
	External collaboration	Interorganizational and cross-sector collaboration	Collaboration	Interorganizational and cross-sector collaboration	Cross-sector collaboration	Cross-sector collaboration	Cross-sector collaboration
Leadership	Leadership	Clear and humble Leadership	Humble leadership and emergent leaders	—	Clear and decisive leadership	Pluralistic governance and adaptive leadership	—

Although studies have attempted to draw lessons from the crisis at different levels of analysis (i.e., organizational vs. health system level), initial insights share several common themes (see Mayo et al., 2021; Reay et al., 2021, for a full overview of literature and topics in health care management literature more generally). Taken together, initial scholarly work during the pandemic points to six main categories that are relevant for health care management both within and beyond crisis: (a) human resources (e.g., training, staffing, staff well-being, retention), (b) operations (e.g., reconfiguration of resources to adjust to shifting demand, supplies), (c) team dynamics (e.g., interpersonal and interprofessional relationships and team climate), (d) external collaboration (e.g., cross-sector and regional collaborations), (e) leadership (approach to leadership, delegation of tasks, decision-making), and (f) innovation (i.e., finding solutions to novel problems, digital tools; see Table 1). Although it is beyond the scope of the present study to offer a full review of these six main categories, they clearly show an emphasis on resources (human, operational, and the need to innovate to optimize limited resources), collaboration (internal via team dynamics and external), and leadership (supporting people, supporting good team dynamics) as essential focus points during crisis.

### The Need for Further Work

As recent reviews have highlighted, the crisis management literature remains scant and lacking empirical studies that evaluate the effects of crisis on organizations (Bundy et al., 2017). Crises not only disrupt organizational life at their most acute stages but also confront organizations with long-lasting changes, challenges, and opportunities for growth. The effects of and required responses evolve over the duration and in the aftermath of crisis (Bundy et al., 2017). Having entered the third year of the COVID-19 crisis, we can thus expect that organizational reality is significantly different than it was in the beginning of 2020. For example, many of the challenges specific to the COVID-19 crisis response and the accompanying high uncertainty in the initial period (e.g., supply insecurity, lack of knowledge about disease spread) are likely to have been resolved or have working strategies in place for resolution. However, it is clear that organizations and professionals are still reeling from longer-term effects brought on by COVID-19 (Hefner & Nembhard, 2021; Muzio & Doh, 2021), and it is important that scholars work to empirically assess what these effects are.

In particular, preexisting challenges are likely to have been reinvigorated (Hefner & Nembhard, 2021) or exacerbated with the onset of COVID-19 (e.g., staff shortages and clinician burnout), but we lack a comprehensive understanding of the challenges that persist for managers now as they move beyond COVID-19 and into the future. With this omission, scholarship also fails to account for how managers may tackle these issues in real time Consequently, proposed strategies may not be as applicable or useful in practice, and management research in the wake of the crisis might remain too far removed from actual practice (Fainshmidt et al., 2021). In addition, health care management literature has failed to consider “grand challenges” (Hefner & Nembhard, 2021) such as COVID-19, leaving a gap in our understanding of (and appropriate responses to) these multifaceted issues. It is thus important that following the onset of such an event, we revisit and empirically assess the challenges facing managers in practice to ensure that our theories remain applicable and responsive to pressing and real-life concerns (Mayo et al., 2021; Muzio & Doh, 2021).

## Methods

### Study Design

We conducted an exploratory case study of hospital management within a heavily affected region of the Netherlands during the COVID-19 crisis. Our level of analysis was the individual (e.g., managers). We chose a qualitative approach based on its appropriateness for gaining a deeper understanding of actors’ experiences—and interpretations of their experiences on the ground (Bluhm et al., 2011). Given the timeliness of the study and a lack of empirical work assessing the challenges currently facing managers inside organizations, understanding how managers currently experience their work and associated challenges is essential to set a base for future scholarship. We spoke to individuals in management and leadership positions across the region to get a diverse perspective on the challenges they face in practice. We focused on the management perspective, trying to understand how the context of the crisis had affected managers in their work, relationships with other stakeholders, approach to management, and what challenges they perceived both now and in the future. Interviews were part of a larger study on hospital adaptation to crisis (Gifford et al., 2022). Ethical approval was granted for this study (FHML-REC/2020/110).

### Setting

As of 2022, the Netherlands has seen five distinct surges of COVID-19 infection rates (National Institute for Public Health and the Environment; RIVM, 2021). The hospitals in our study are all located in one of the most heavily hit regions by the pandemic. There are five hospitals in the region that vary in size (e.g., in terms of annual turnover, number of beds, and amount of staff) and of which one is an academic hospital, two are top clinical hospitals, and two are general hospitals (see also Gifford et al., 2022). The academic hospital, the two top clinical hospitals, and one general hospital have been included in this study. Like all hospitals in the country, the hospitals we studied are private nonprofit organizations of which the services are (selectively) contracted by health insurers.

### Data Sources and Collection

We conducted two rounds of interviews. Considering the lack of empirical work focused at the level of HCOs during the pandemic and in attempts to answer calls to bring the voices of managers and leaders forward (Aguinis et al., 2022), interviews were chosen as an appropriate method to capture the persistent challenges. Interviewees were invited to participate in a first round of interviews in September to December 2020, which coincided with the second wave of infections. They were initially approached by a liaison appointed in each participating hospital who helped us gain access in a way that minimized additional burden on the organization during the pandemic. The liaison provided us contact details of individuals willing to participate, whom we subsequently contacted by e-mail. All interviewees provided informed consent for interviews. Because of social distancing measures, the majority of interviews were conducted online using video conferencing software. All interviews were recorded and transcribed, and detailed summaries were made following each interview. Each participant received a summary of their interview for member checking.

In the first round, we interviewed 23 interviewees who had management roles. They included board members, managers (including human resource [HR] managers and department heads), and medical leaders (e.g., medical directors and clinicians with management roles). Topics in this initial exploratory round focused on how the organization adapted to the crisis. We reserved a portion of these interviews for individuals to reflect upon the challenges they faced in their roles as managers and leaders and thoughts on future challenges and their own functioning during the crisis (see Supplemental Digital Content for interview guide, http://links.lww.com/HCMR/A112). Roughly 1 year later, we invited all 23 interviewees to participate in follow-up interviews. Interviews took place between July 2021 and December 2021, coinciding with what has now been called the fourth wave of infections. Ultimately, 12 interviewees from the first round, across four organizations, were available for a follow-up interview. In addition, five new interviews were conducted to supplement limitations of the first sample (e.g., management in H4 and medical leaders). The sample in the second round thus consisted of 17 interviewees. Table 2 shows the interviewees included per hospital for both Round 1 (R1) and Round 2 (R1).

**Table 2:** Data per hospital

Round 1	Board	Management	Medical leader	Totals	Round 2	Board	Management	Medical leader	Totals
H1	2	5	1	8	H1	2	3	2	7
H2	2	3	2	7	H2	2		1	3
H3	1	3	2	6	H3		2		2
H4		1	1	2	H4		4	1	5
*Totals*	5	12	5	23		4	9	4	17

Interviews in the second round were guided by a topic list that was created from analysis of the first round of interviews (main themes identified were: HR challenges, balancing of priorities, dealing with uncertainty, and leadership (in crisis) and informed by ongoing research and interview studies throughout the year (see Supplemental Digital Content for interview guide, http://links.lww.com/HCMR/A112). Interviewees were asked to reflect on their experience of managing the second year of the crisis and whether they experienced differences and similarities between the first and second year, what the crisis has taught them about managing a hospital during (prolonged) crisis, and whether they foresaw challenges for management going forward (for the remainder of the crisis, but also the years following that). Importantly, in this second round, we were able to go beyond the initial crisis response and ask interviewees to reflect upon the lessons and challenges that remained salient and pressing.

### Analysis

We took an interpretive approach to data analysis (Stake, 1995), working from the data in order to gain insights and induct insights from practice. Given the nature of the data collection, analysis can be seen as organized into two stages. The first round of interviews were analyzed iteratively, and interview guides and topics were updated as we collected more data in order to capture salient themes. Interviews were analyzed with a focus on lessons learned during crisis, ongoing challenges, and future challenges. In this initial round of analysis, we coded the data using thematic analysis (Miles & Huberman 1994) and identified several themes that were salient for follow-up, including HR challenges, balancing of priorities, dealing with uncertainty, and leadership in crisis.

Once the second round of interviews were collected, we worked to analyze each set separately again, starting with the first round to identify the central challenges identified by participants. We then did the same initial round of thematic analysis for the follow-up interviews. From here, we engaged in constant comparative analysis (Glaser, 1965) to highlight relevant changes (e.g., we saw the emphasis on staff shortages as a challenge emerged much more significantly in the follow-up round and that emphasis was less on crisis leadership than on leadership more generally) and differences or similarities between the two time periods. Our interest was ultimately in unveiling the *persistent* issues facing management, and therefore, in a final step, we identified persistent challenges as the challenges that were exacerbated or brought to the forefront by the crisis that continue to face management now and are likely to play a considerable role in the years to come. Using both rounds of interviews allowed us to see what issues were crisis specific (e.g., indicated by their salience in the first wave, but resolution by the second wave) and what issues were indicative of persistent challenges (being exacerbated by the crisis and lingering still). After identifying the central challenges, we compared the persisting challenges in relation to the categories abstracted from the literature to see how our empirical setting translates and to create an agenda for health care management going forward, which we present in the discussion chapter (see Table 3).

**Table 3:** Coding tree

First-order codes	Second order	Third order	Key challenges
Absenteeism Retention difficulties Recruitment challenges Turnover	Staff shortages	Human resource issues	The centrality of human resource constraints amidst high demand
Mental strain Physical strain High workload Feeling undervalued Lack of appreciation	Burden on staff		
Balancing regular and COVID-19 care Disease complexity Patient backlog due to postponed care Waitlists	Increasing demand	Financial constraints and high demand	
Budget constraints Concerns over future budget Top-down budget The need for prioritization Hiring freeze	Financial constraints		
Common enemy/shared goal High willingness and need to cooperate Patient flow collaboration Collective procurement Information sharing Utility of strategic partnerships Regional decision-making Limited resources and high demand	[Need for] external collaboration	External collaboration	Needing collaboration, driven by competition
Inherent competition Compelled to protect organizational autonomy Financial independence System enforced boundaries Competing demands Financial incentives	Barriers to collaboration		
Quick decision-making Streamlined structures Leading top-down People want firm decisions	The need for decisive action	Governance and leadership	Tensions in leadership: decisive versus inclusive
Broader inclusion of staff Need for diverse perspectives Responsibility at all levels Bottom-up management Autonomy of staff	Importance of inclusiveness and autonomy		

## Findings

Beyond the specific challenges that emerged as a result of the pandemic, participants identified several persistent challenges facing them in their work. These include challenges relating to resources and demand (human and financial resource constraints in the face of increased demand) collaboration, and leadership and governance. We detail these challenges in the following sections.

### Resource Constraints Amidst High Demand

#### Human Resources

A central challenge mentioned by all interviewees concerned a shortage of human resources, in particular nurses. In the first wave, interviewees raised concerns about staffing, but the focus was mainly on reconfiguration of staff, protection, and redeployments. Having gone through the COVID-19 period 1 year later, our interviewees were able to reflect upon the grander challenge of staff recruitment, retention, and turnover that has been exacerbated by the demands of the COVID-19 period.

Staffing, it’s always an issue in the cure as well as in the care. It’s a nationwide problem. I think it’s even a European problem. It’s always critical, but the things that are happening now, the last months, it started I think about April or May when you really saw that there was a difference in the attitude and the effects that it had. Now, it’s clearly stronger than ever. It’s always been a problem, but it was a manageable problem. Now, it’s an effect which we are struggling with, to be honest. (Manager, H4, R2)^1^

Shortages were an ongoing issue prior to COVID-19, but during the first wave of the crisis, the additional demand for COVID-19 care could be met because regular care was stopped. This allowed staff from other functions to free up and support in areas with limited staff such as the intensive care unit (ICU). When hospitals began resuming regular operations and care delivery, it became clear that the capacity of staff to handle the high demand was severely restricted. Even without high COVID-19 demand, management struggled to keep beds open and to meet ongoing demand due to staff shortages (which were exacerbated due to high absenteeism of staff), and deciding between lowering the burden of staff and meeting demand proved an ongoing challenge.

When I talk to the nurses in the department, the management tells them to close beds, so that’s a way to lower the work pressure on nurses if you don’t have enough nursing staff. Then again, there’s another force telling them to open beds because patients keep coming in, and the hospital is filled. In the X department, for example, they have six closed beds. Then in the evening, there’s always a manager who will call them and say, you have to open beds because there are patients in the emergency department. The beds are there, but then the nursing staff says, “yes there are beds but there’s no nursing staff.” The managers then tell them, “open the beds.” Next morning, too many beds are open, and not enough nursing staff. They work hard, but you can imagine that pressure rises, and then they will get burnout, sick, and they lose faith in the management. All in all that’s not a very good way to go. (Medical leader, H1, R2)

All but one interviewee indicated that the workload and stress of COVID-19 only worsened this already growing problem (of shortages). Interviewees commented on the fact that the workload, pressure, and the difficult physical and emotional nature of the work (especially for nurses) eventually take its toll on staff. In Round 2 of interviews, management described turnover as a serious and growing concern. Although shortages were present prior to COVID-19, interviewees described the crisis as a sort of “tipping point” for what was already a growing issue with retaining existing staff and recruiting new staff, particularly in areas such as ICU. Many interviewees spoke of nurses leaving the profession (altogether or via early retirement), the hospital sector (e.g., to nursing or home care), and the organization (to another hospital).

Most of the nursing staff are ill; the others have searched for other jobs. They will not be working at the hospital anymore. They want to do different things, everything but work at ICU. There are a great number of nurses who work at the IC, who will give signals that they are very tired, and well, they have not reported illness yet but they are very close to it. It’s very difficult to get the staff for the necessary beds. (Medical leader, H2, R2)

This concern extended beyond the COVID-19 period and includes an ongoing challenge of recruiting new staff, particularly in intensive areas such as IC units.

#### Financial constraints

Hospitals operate in resource-constrained environments, and management must constantly balance priorities in order to work within financial constraints amidst increasing demand. However, the demands on the health care system are not new. Many interviewees, especially those in more strategic positions, highlighted the fact that the system has been pressed for some time. Although operational, HCOs have been working at the top of their limits, coping with an aging population, increased complexity of disease and chronic and multimorbidities. The primary challenge with these environmental shifts is that while demand grows, budgets do not increase at the same rate or remain stagnant. With the onset of COVID-19, the extent of this challenge came to a head, and the crisis revealed how rigid the system had become due to ongoing constraints. Interviewees recognized that when demand increases significantly, there is no longer room in the system to adjust.

For me the biggest challenge is, what will COVID do? And how will we, in our normal, regular capacity, create space for that. But that all has to do with financing. And that sounds very stupid, but unfortunately our system revolves around money. So when no one wants to pay for it, no capacity will be created…[and] everybody wants to [create extra capacity]. I mean the hospitals are willing, I am willing, to generate an additional 20 beds and do catch-up care during the summer and COVID care during the winter, but someone has to pay for that. (Manager, H3, R2)

For management, financial constraints pose a persistent challenge that limit their scope of action and, particularly in periods of increased demand, creates dilemmas. Although constraints are usually considered at a higher, system level, we find that the effects filter down and have significant effects on the management of day-to-day practice and operations within organizations. In extreme contexts, such as the COVID-19 pandemic, the reality of these constraints come to directly impact management’s scope of action, for example, when management must prioritize between two “essential” aspects such as hiring more staff or implementing a hiring freeze to reduce costs. For management, resource constraints imposed from above directly impact their ability to act, which forces them to act as so-called “middle men” between higher-level constraints and the working floor. This means that management take on the front-facing role of top-down decisions, for example, having to deny new projects, reject business plans, halt the hiring of new staff, or deny the purchasing of new equipment.

We have to reduce the number [of staff] because now we’re digitizing, automating the total process. […] It is also challenging because you have people who work 30, 35 years or 40, so you have to make good decisions. So you have to replace or go and look for other jobs for them, or you have from now until your retirement you can still work here. But when you leave, we will not fill in the gap. That’s another challenge. (Manager, H1, R2)

At a higher level, constraints mean needing to take more drastic measures and prioritize between, for example, innovation and practical concerns, such as expanding existing wards.

### Collaboration

During the crisis, collaboration with external partners intensified. This was due to necessity and urgency of conducting better triage (e.g., the general practitioner taking a larger role), transferring patients, handling patient spread (e.g., centralized capacity planning), and procuring supplies. Hospitals worked together in the region to support one another, share information, create plans of action, and consult on decision-making. Regions collaborated to spread patients and secure supplies, and sectors worked together to support the crisis response. In a way, the crisis helped to remove the persistent barriers to external collaboration that exist, showing a way forward.

Maybe we could learn from this pandemic that it has many advantages to work more together with other hospitals, before the Covid crisis, we didn't work so much together. It was each hospital for its own. It was more the competitive model and the more neoliberal model, but I think now many hospital boards are more eager to work together and also to have some national, organised national measures…I think many doctors are not very convinced about this…. But I think that has more to do with the whole financial organisation of hospitals, that should also change […] we have to organise the health care different than we have done until now. (Medical leader, H4, R2)

Unfortunately and as our interviewees indicated, barriers remain an issue to fostering better cross-sectoral and interorganizational collaboration. This includes financial barriers that induce not only competition but also a tension between organizational interest and interests at a collective level. The challenge for management included knowing when to balance organizational autonomy with the need for a shared sense of responsibility and centralized decision-making. In addition, those in more strategic positions commented that the continued presence of competition at a system level continues to pose a complicating factor for fostering collaboration and teamwork.

Yes, there are still elements of competition. […] But there is also more working together. That is what I see, especially at this time, we have too many patients that are waiting, we really try to help each other in the hospitals. […] So when [hospital X] has two operating rooms free on Friday, they call patients and doctors from our hospital in order to do their procedure. So we help each other and we help the patients, of course, and there is much more working together, but there is still competition. (Board member, H1, R2)

They commented that competition in the system has made it difficult to engage in the necessary collaborations without sacrificing the needs of the organization in terms of financing and having a competitive advantage. Competition was mentioned mostly between the same organizations (e.g., hospitals), and financing was listed as an additional ongoing challenge to shifting care and collaborating more with the first line (general practitioners).

### Governance and Leadership

A challenge faced by hospital leadership during the COVID-19 period was the governance of the organization. In the beginning, organizations all shifted into a crisis management structure, creating crisis teams and operational teams to make decisions and policies. Although these structures were deemed successful and necessary as it resulted in quick and strategic decision-making, as the crisis lingered, several issues remained in regard to governing in the context of environmental uncertainty. In particular, management recognized the ongoing tensions between the need for quick, decisive action and the slower but more inclusive process of decision-making with involvement of all groups.

An additional challenge was how to make necessary changes and take swift action without losing the clarity of leadership. All interviewees recognized the need to be able to react quickly to environmental changes and to address uncertainty, and during the crisis, this often meant removing some key players and streamlining decision-making power. However, as leaders recognized, if in the process of streamlining decision-making the governance structure becomes unclear, communication can break down and introduce more complexities.

We skipped all the others [layers]. They were no longer important. That was a belief at that time that that was necessary. But we learn now this is not smart to do because from the first point, communication is no longer functioning because nobody knew who is my manager, who can I talk to? That was no longer clear…. And getting the right things done was a very complex process. So decision making was OK. But then getting the things done was again more complex because nobody understood how the structure worked. (Board member, H2, R1)

In light of the struggle between balancing quick decision-making while involving others and remaining clear structures, interviewees commented on the need to rethink the overall governance structure going forward, whereby increased autonomy of health care workers and bottom-up management were deemed important aspects to consider.

Common goal, it’s the same thing [as a common enemy]. I think as a manager, you have to explain the goal and then ask them, can we work together to reach the goal? Because thinking about solutions as a manager, it’s always spreadsheet management. It never works in practice like that…I think it works better if the staff member on the work floor thinks about the solution and then you work it out together. (Medical leader, H1, R2)

Although interviewees acknowledged the need for more autonomy and bottom-up management, what future governance structures should look like and finding the right balance between leading and delegating were considered continuing challenges.

## Discussion

Our discussion is divided into two parts. First, we bring forward the key persistent challenges identified in our empirical data and situate these challenges within the current literature. Second, based upon this synthesis, we highlight two key areas for future research. Initial literature identified three overarching themes relevant for health care management amidst the COVID-19 pandemic: resources, collaboration, and leadership (see Table 1). Although these themes remain salient, our study adds important contextual differences and offers a central contribution by putting a spotlight on three persistent challenges facing health care managers beyond the crisis: the centrality of (human) resource constraints (amidst high demand), the necessity of collaboration (amidst competition), and reconsidering the approach to leadership (humble versus decisive). These challenges are interconnected and salient issues that will continue to face managers and organizations in the coming years, requiring novel approaches.

### Health Care Management in the Wake of a Crisis: Persistent Challenges

#### Recognizing the root cause: human resource constraints

From our data, it is clear that organizations face increased demand while their resources remain relatively stagnant (e.g., finances) or decrease (e.g., human resource dropout). Interestingly, we find that for our interviewees, in the wake of the crisis, operations become less of a focal point than has been explicated in previous work. The focus on capacity planning, beds, and even supply uncertainty significantly shifted over the course of the crisis. Although operational issues persist, our study indicates that operational challenges in the present moment are primarily a symptom of human resource issues. This echoes the sentiments of other scholars who suggest that COVID-19 has shined a light on the importance of human resources and management (Collings, McMackin, et al., 2021; Collings, Nyberg, et al., 2021). Although financial resources play a significant role in directing and constraining decision-making, human resource shortages create new and significant challenges in the area of operational management and service delivery. For example, capacity planning must take into account availability of human resources to determine the amount of beds that can be opened with a safe ratio of staff. As a result, our data indicate, in line with other studies (Begun & Jiang, 2020; Forman et al., 2020; Nembhard et al., 2020), that the need for creative solutions regarding how to handle capacity (and meet demand) is present. It also becomes clear that the need to “put people first” remains a focal point and challenge for the future, aligning with the emergent work on the well-being of health care workers (Alami et al., 2021; Begun & Jiang, 2020; Nembhard et al., 2020) and importance of workforce retention (Hefner & Nembhard 2021)

#### Driven by competition, in need of collaboration

In line with other literature, collaboration emerged as a central theme in our data. As Muzio and Doh (2021) point out, COVID-19 has “exposed and reinforced” the multilevel and cross-sector interdependencies that exist within our current world while at the same time highlighting divisions. In health care, this interdependence is more exacerbated as organizations operate in an increasingly resource-constrained environment (Reay et al., 2021) and health care delivery becomes more complex and multidisciplinary. However, although our data indicate that collaboration is becoming increasingly necessary for organizations to remain responsive to environmental demands (Alami et al., 2021; see also Mayo et al., 2021, for a full review), competition remains a driving force in many systems as a means to improve efficiency and consumer choice. Financial incentives coupled with resource constraints work to drive competition between sectors, organizations, and even units within organizations (Powell & Davies, 2012). This reality lends to a gap between theorized strategies (e.g., fostering collaboration) and practical constraints (e.g., perverse incentives). For management, this manifests in institutional constraints that limit the scope of action and creates barriers to the interorganizational work needed to meet environmental demands. As Mayo et al. (2021) highlight, competitive forces in health care can limit the ability of HCOs to enact necessary changes, and that attention to the broader environment in which HCOs are situated (e.g., operating in competitive environments) is lacking in current health care scholarship.

#### Leadership: decisive versus inclusive

Previous studies emphasized the importance of what was termed “humble leadership” (see Schein & Schein, 2018, for a full overview). This approach that focuses on “bottom-up” leadership (Owens & Hekman, 2012) calls for recognition that those in the organization, often with less power, also have an important contribution to make. As this approach to leadership assumes, leaders cannot function alone and should recognize “every worker as having the capacity to identify and solve problems” (Nembhard et al., 2020, p. 5). In general, our data support the notion that a humble approach to leadership can better support organizational goals and meet pressing demands. Empowering staff to take part in problem solving and advancement from the ground up helps circumvent the negative sides of top-down management, especially when working with knowledge professionals who are traditionally resistant to this style (Powell & Davies 2012). However, leaders did express feeling a tension between what they initially perceived as a need for quick, decisive action (which they often undertook in a more command and control style) and later recognition that they should have better delegated tasks and included staff in decision-making.

Humble leadership requires leaders to be willing to admit mistakes and limitations, acknowledge ongoing uncertainty, and spotlight the strengths and contributions of staff within the organization to support organizational goals (Owens & Hekman, 2012). When leaders in our data were able to admit their shortcomings in early decision-making, for example, being too short-sighted or not involving enough staff, they also were able to effectively model “teachability,” which can support a more open culture (Owens & Hekman, 2012). In this way, humble leadership can inspire and encourage mutuality, whereby all stakeholders and organizational members align on a common vision in their work to “coproduce health care” (Howieson et al., 2013) and generate effective solutions to persistent problems. However, humility in leadership does not imply a lack of decisiveness. Rather, in times of crisis, decisiveness is key but should be carefully balanced with acknowledgment of others’ expertise, bottom-up decision-making where possible, and recognition of one’s own limitations.

### Setting a Future Research Agenda

In consideration of the persistent challenges facing health care management in the wake of the COVID-19 crisis, we formulate two key areas of research that can support further exploration of challenges in practice and expansion of theory in the health care management domain. First, drawing upon the emergent literature during COVID-19 and our present findings, we emphasize the “human” nature (Collings, McMackin, et al., 2021) of the issues facing management postcrisis and look to the literature on professional work to draw useful insights for health care management in practice. Here, scholars may draw upon and expand literature on professional labor and managing professionals (Freidson, 1988; Yanchus et al., 2020) and consider the applicability of shared decision-making models to clinician and management relations. Second, as scholars have noted, the pandemic acts as a disruptive event that can increase the salience of paradoxes and complicate organizational responses (Carmine et al., 2021). Ultimately, our data support this notion and point to the utility of applying insights from paradox theory to support managers and organizations in tackling these persistent issues.

#### People work: emphasizing the need for bottom up management

Our data highlight that, within each central issue, understanding the needs and desires of and interconnections between individuals is central. This aligns with scholarship in the beginning of the crisis that emphasized “putting people first” (Nembhard et al., 2020) and human resource management scholars who have signaled that the COVID-19 crisis is largely a “human one” with human resources and HR management at the center stage (Collings, McMackin, et al., 2021; Collings, Nyberg, et al., 2021). In particular, the importance of valuing and supporting workers (Hefner & Nembhard, 2021; Hick & Biddinger, 2020) and workers’ involvement in organizational decision-making remains a focal point as organizations move beyond the crisis. This echoes the sentiments of a humble, bottom-up approach to leadership, which was emphasized in emergent literature and present in our data and has gained increasing attention in the scholarly literature more broadly because of its importance for managing in more uncertain and unpredictable environments such as the ones HCOs face today (Owen & Hekman 2012).

##### Managing professionals

In health care, literature on professional labor and managing professionals (Freidson, 1988; Yanchus et al., 2020) may offer useful insights (and raise important questions) for researchers in the postpandemic world (Hoff, 2021). This aforementioned body of literature has detailed the importance of valuing the tacit knowledge and skills of physicians, the well-being and human resource benefits of granting physicians high levels of autonomy and influence (Hoff et al., 2021), and how to manage and work with this unique type of worker (Reay et al., 2021). Our results reveal that these factors may also pertain to a broader group of occupational groups, for example, the benefits of granting nurses and residents more autonomy and responsibility were shared clearly throughout the crisis. A shortcoming of the application of this professional literature in health care is thus the emphasis on physicians at the expense of considering the benefits of this type of management approach for other skilled workers such as nurses that become increasingly professionalized. In future research, beginning to align the perspectives and expertise of a diverse set of skilled workers is essential, and staff involvement constitutes a key part of organizational decision-making in HCOs (Alexander et al., 2007).

##### Shared-decision making

Our study highlights that cultivating understanding for the realities and challenges of work must be bidirectional, from management to the work floor and from the work floor toward management. As health systems move forward in the coming years, it will become increasingly important to foster understanding from the work floor of the realities of the challenges facing management (see Noordegraaf, 2011) and for management to include staff in strategic decision-making. Recognition of the impossibilities facing health care systems and thus managers inside organizations can support shared decision-making and buy-in for strategies. Although shared decision-making is a model most used in the professional–patient relationship (e.g., Issel, 2019), its main tenants align with a more humble approach to leading by sharing decision-making power and empowering professionals to make decisions (Schein & Schein, 2018). Moving forward, it is interesting to consider how shared decision-making models could be applied to the management and health care professional interaction. In our study, managers found that coupling involvement in decision-making with responsibility and accountability for decisions enabled buy-in and support for decisions on the longer term (see also Alexander et al., 2007). Future research might consider more inclusive models of decision-making that also support a sense of accountability for the trajectory of decision-making, including discussion, deciding, implementing, and outcomes.

#### Finding possibilities in impossible times: a paradox lens to health care management

A hallmark of crisis (management) is the presence of uncertainty. Because of high uncertainty, management are forced to make difficult decisions with limited information and face significant dilemmas and paradoxes in their work (Collings, Nyberg, et al., 2021). Paradox is conceptualized as “contradictory yet interrelated elements that exist simultaneously and persist over time” (Smith & Lewis, 2011, p. 382). Such dilemmas often require trade-offs, for example, stopping regular care to increase capacity for crisis care. However, our data indicate that dealing with paradoxes has become a new normal of health care management both presently and into the near future (see also Issel, 2019), with inherent tensions present in all persistent challenges. Specifically, we find that organizations struggle between high and complex demand versus financial operational and human resource constraints, collaboration versus competition, and autonomy and inclusive decision-making versus top-down and decisive leadership. This requires a new approach to management that encapsulates the need to meet competing and often contradictory demands (a “both-and” approach) as well as recognizing where trade-offs can be made (“either-or” approach) and emphasizes the importance of prioritization.

##### Responding to paradox

Paradox research offers some insights into the situation facing management in practice. Paradox theory has been widely researched in the fields of general and strategic management and organization theory (Cunha & Putnam, 2019; Smith & Lewis, 2011) but to date has had limited application in health care management (see Issel, 2019; Peirce, 2000, for exceptions). However, recently, scholars have called for a more dynamic exploration of paradoxes in practice and of other types of responses to paradoxes. Cunha and Putnam (2019) suggest that a combination of both-and and either-or responses may be relevant, depending on the type of paradox, and in health care, unique responses may be required. This resonates with our empirical findings; for example, our interviewees indicated that the both-and response to collaboration and competition, often cited as “coopetition” (Westra et al., 2017), has failed to function in practice. The data suggest rather that an “either-or” approach (e.g., cooperation or competition) is most appropriate for this context, given the needs of the system and the clients and professionals at the center. This example indicates that approaches to paradox may not always be about embracing opposing elements and may require decisive action and admittance of error or failures at a higher level (e.g., competition failing to deliver or working in contradiction to health system goals) to meet the needs of clients.

Future research should work to uncover the central paradoxes, and types of paradoxes, facing managers in practice in order to explore what strategies are best suited and available for management in resource-constrained environments. This is important as each type of paradox may require a different management style and approach. Consideration of situational determinants (Strasser, 1983), including both external and internal constraints, will impact how management approach each situation and should inform future scholarship. Studies that incorporate assessment and identification of paradoxes and active management approaches tailored to resolve or exploit tensions for the benefit of organizational goals would be particularly salient.

### Limitations

The present study allows us to reveal the persistent challenges facing health care managers and helps us to move beyond the context of COVID-19 in order to map a future research agenda for health care management. However, there are some notable limitations that may be addressed by future studies. First, the present study lacks engagement with those working on the frontlines, who are arguably most affected by management decisions and approaches. Incorporating the perspectives of both frontline staff and management into future research as scholars work to offer practical recommendations would help to offer holistic insights and strategies. Second, this study excludes consideration of other types of HCOs (e.g., nursing homes, rehabilitation centers, etc.) and focuses on one country context with specific financing models and regulations. It thus remains unclear if our findings translate to these other organizational and national settings. However, the challenges we present here are likely to exist in some capacity across the health system due to their persistent and systematic nature. Future research may incorporate comparative work to consider how the stated challenges translate to other national contexts and discover important contextual factors.

### Practice Implications

Although the COVID-19 crisis introduces a host of new challenges for health care systems, it also unveils weak points that were already present within the system. First, at the core of persistent challenges are largely “people” issues that require a humble and human approach. This means that managers and leaders must work to move toward a humble style of leading, where there is openness about fallibility and opportunities for meaningful input in order to create an environment in which staff feel heard, empowered, and appreciated. For instance, team leaders can support a caring work climate (Rathert et al., 2022) by listening to their staff, scheduling frequent check-in moments with team members, and offering regular opportunities for input from the team. Such a process should also then filter up as team leaders or managers have opportunities to feedback to leaders and offer input directly from staff.

Second, management confronts inherent tensions between essentials as they face resource constraints coupled with increased (and sustained) demand. This calls for an emphasis on prioritization and informed decision-making, and fostering a mutual understanding of the constraints on management and organizations to avoid dissatisfaction of staff. For example, building in more open discussions between managers, staff, and hospital leadership, along with a humble approach to leading, can allow management and leaders to speak candidly about the challenges they face. Open forums where the realities of internal constraints (e.g., resources) are discussed and are opened up for input about solutions can foster a sense of communal responsibility in facing issues of scarcity and support bringing worlds together toward solutions. In larger organizations, electing internal committees made up of staff from multiple organizational levels and functions may be one way to support generating creative solutions to persistent challenges and shared decision-making.

## Supplementary Material

## Footnotes

^1^
Where necessary, quotes have been edited to preserve anonymity and for clarity. Interviewees are denoted by their affiliated organization (H1, H2) and corresponding interview round (R1 = first round, R2 = second round).

## Interview Guide Round 1

**Orientation**

Hospital information, history/background, structure.

Organizational structure

Cultural aspects

**Opening**

Could we please begin with you describing your current role? If you could also describe what role have you played specifically in the organizational/regional/departmental response to COVID-19?

**COVID-19 response**

Retrospective: Could you please shortly walk me through how the organization first became aware of and responded to the COVID-19 pandemic?

Decision-making structure: Who was/is in charge of determining response? How were/are decisions communicated and agreed?

What was your level of involvement in this response/adaptation? Were you given the opportunity to be involved?

When did the organization begin its response to the COVID-19 crisis?

Could you please describe how the organization/your department has responded to the COVID-19 crisis?

What adaptations had to be made? (If multiple adaptations, try to establish a timeline and prioritization)

**Effects on work:**

How has the situation affected you personally in your work?

Could you please describe the effects of any adaptations/responses? On the content (e.g., tasks)/structure (e.g., timing) of your work? On organizational functioning?

What was done to safeguard workers? Physical health? Mental/emotional health? Fatigue? Recovery opportunities? Do you perceive any long term effects?

Were workers able (i.e., did they have the knowledge, skills, abilities, other relevant characteristics) to cope with demands? How were they supported in the transition?

Prior to COVID-19, were there any staffing issues (shortages/workload/issues)?

If so, how were these handled during the crisis? Impacted?

*Professionals:*

Did you feel able to carry out your work? The work you were being asked to do?

Did you have adequate resources? (personal, material, physical, emotional) If not, what would you have needed to function appropriately? What was missing?

(How) were you facilitated by the organization in carrying out your work?

How did/do you balance work/leisure time?

What has been your experience of work before, during, and after the first wave? Was your satisfaction affected? Your capabilities/functionality?

What do you expect in terms of long-term effects on your ability to function at work following COVID-19?

How have your health (e.g., physical, psychological, fatigue), well-being at work (e.g., motivation and satisfaction), competences, performance, and chances on the (internal/external) labor market been affected by COVID-19 and the adaptations in your hospital?

**Dynamic capabilities**

How did you feel about the organizational response more generally?

Are there things you would have done differently?

What do you think the organization did well? Could have been done better?

What enabled/prevented the organization in their adaptation

What capabilities do you think hospitals need to respond to crises such as this one?

Do you see differences with other hospitals in terms of response? Prioritization?

**Interorganizational dynamics/regional level**

What was the role of org X in terms of coordinating a regional response?

Did you work together with other hospitals or organizations? (Was this necessary/voluntary?)

Do/did you need support from/support other organizations?

What about the collaboration with other sectors? With ROAZ (Regional Network Acute Care)?

## Interview Guide Round 2

**To get started:**

Has your work or role changed at all since we last spoke?

What is the status of COVID-19 care in the hospital?

Current capacity and regulations regarding care distribution (regular/COVID-19 care)

Are you taking in transfers/transferring out?

What are your thoughts on such centralized/collective programs (e.g., collective slack)?

Human resources

How are your staff/colleagues currently coping with the demand?

Are you experiencing any shortages of staff? If so, what causes these shortages?

How is the current collaboration between teams/functions?

Are you experiencing difficulties deploying staff for certain care (e.g., COVID care)?

Have you changed your approach to HR since we last spoke? Do you have plans to?

**Workload**

How has your workload been throughout the crisis? Has this changed significantly? If so,

how?

Decision-making—how has this changed from your position?

**Organizational structure**

What are the main difficulties you experience in your work?

What emotions have you experienced since working during COVID-19?

**Support/recovery**

Have you had opportunities for recovery?

Are you able to detach from your work?

How do you recover in your free time?

Has your ability to recover/relax been impacted by COVID-19?

**Reflection**

Are there any things you would have done differently?

In your opinion, are there things that could have been handled better? (by the organization, government)

What have you learned about being a manager through this crisis? What has the crisis taught you about leadership?

What is unique about this situation/what is generalizable?

What opportunities has this crisis brought?

What changes will remain?

How are you tackling the human resource issue?

Have you changed strategies in regard to material goods/org structure?

Do you feel better prepared to deal with crisis/tackle uncertainty as a manager? As an organization?

What are the biggest challenges you face right now and in the next years? As a leader/organization?

What are the biggest challenges facing health care field in general?

Looking forward, do you see any changes in the health care field as result of the crisis?
